# Automatic detection of AutoPEEP during controlled mechanical ventilation

**DOI:** 10.1186/1475-925X-11-32

**Published:** 2012-06-20

**Authors:** Quang-Thang Nguyen, Dominique Pastor, Erwan L’Her

**Affiliations:** 1 Department of Signal and Communications, Institut Télécom; Télécom Bretagne, 29238 Brest, France; 2 Medical Intensive Care Unit, CHRU de Brest/INSERM U1101 Latim Ubo, 29200 Brest, France

**Keywords:** Patient-ventilator interaction, Dynamic hyperinflation detection, AutoPEEP detection, Signal norm testing, Sequential decision

## Abstract

**Background:**

Dynamic hyperinflation, hereafter called AutoPEEP (auto-positive end expiratory pressure) with some slight language abuse, is a frequent deleterious phenomenon in patients undergoing mechanical ventilation. Although not readily quantifiable, AutoPEEP can be recognized on the expiratory portion of the flow waveform. If expiratory flow does not return to zero before the next inspiration, AutoPEEP is present. This simple detection however requires the eye of an expert clinician at the patient’s bedside. An automatic detection of AutoPEEP should be helpful to optimize care.

**Methods:**

In this paper, a platform for automatic detection of AutoPEEP based on the flow signal available on most of recent mechanical ventilators is introduced. The detection algorithms are developed on the basis of robust non-parametric hypothesis testings that require no prior information on the signal distribution. In particular, two detectors are proposed: one is based on SNT (Signal Norm Testing) and the other is an extension of SNT in the sequential framework. The performance assessment was carried out on a respiratory system analog and *ex-vivo* on various retrospectively acquired patient curves.

**Results:**

The experiment results have shown that the proposed algorithm provides relevant AutoPEEP detection on both simulated and real data. The analysis of clinical data has shown that the proposed detectors can be used to automatically detect AutoPEEP with an accuracy of 93% and a recall (sensitivity) of 90%.

**Conclusions:**

The proposed platform provides an automatic early detection of AutoPEEP. Such functionality can be integrated in the currently used mechanical ventilator for continuous monitoring of the patient-ventilator interface and, therefore, alleviate the clinician task.

## Introduction

Mechanical ventilation is routinely used in the clinical ward and/or in nursing/rehabilitation institutions. Unfortunately, imperfect interaction between patient and ventilator is frequently exhibited in intubated patients [1] and those undergoing non-invasive ventilation [2].

It has been demonstrated that the graphical curves (flow, airway pressure and air volume) available on most recent mechanical ventilators provide much information to analyze the patient-ventilator interface [3]. By visually monitoring these curves, patient-ventilator mismatching can be observed and detected by the clinician. Various automatic detection algorithms either embedded in a ventilatory system to detect ineffective triggering and double triggering [4], or recently in a computerized monitoring system (BetterCare) to determine ineffective respiratory efforts during expiration [5] have been reported with positive results. However, to the best of our knowledge, the automatic detection of other types of ventilatory abnormalities, including AutoPEEP, has not yet been adequately considered.

This paper addresses automatic detection of AutoPEEP, a common ventilatory abnormality that usually occurs in patients with acute severe asthma or chronic obstructive pulmonary disease. The presence of AutoPEEP basically indicates an insufficient expiratory time. The amount of time given over to expiration therefore needs to be lengthened, either by reducing the respiration rate or by decreasing the inspiratory time, or both. AutoPEEP can be measured at the patient’s bedside by using the pressure transducer of the ventilator. However, this quantification requires intervention from the therapist, who must perform an expiratory pause, in order to monitor tele-expiratory pressure [6]. On the contrary, although not readily quantifiable, AutoPEEP can easily be recognized on the expiratory portion of the flow waveform. If expiratory flow does not return to zero before the next inspiration, AutoPEEP is present. This detection however requires the eye of an expert clinician at the patient’s bedside. Using flow signal as the input, an automatic detection of AutoPEEP (dynamic hyperinflation) due to either expiratory flow limitation and/or inappropriate ventilatory cycling should be helpful to optimize care. Our focus is thus early detection of AutoPEEP for continuous monitoring of the patient-ventilator interface. In what follows, AutoPEEP detection is performed by Signal Norm Testing (SNT) on the flow signal captured from the patient-ventilator interface. SNT involves testing the norm of a signal observed in noisy condition with respect to a certain tolerance fixed by users on the basis of their know-how and/or experience of the domain [7]. An extension of SNT in a sequential framework is also investigated. Other practical aspects, including phase change detection and parameter estimation are considered as well. The performance assessment is provided in three levels. First, the detection performance of the proposed detectors will be illustrated with data synthesized on computer. Then, further evaluation is performed on data derived from a respiratory system analog. Finally, an *ex-vivo* performance assessment on retrospective data acquired from patients is carried out.

## Methods

### Automatic detection of AutoPEEP and System overview

**Figure 1:**
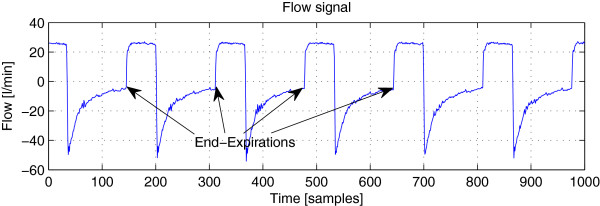
**An example of flow signal.** This signal was recorded during the assisted mechanical ventilation on a patient. The (blue) curve shows a typical waveform of flow signal with squared inspiratory phase. The arrows point to some end-expiration instants where the markers for AutoPEEP detection are present.

AutoPEEP can be visually observed and detected through flow signal. Figure 1 shows an example of flow signal with AutoPEEP captured during mechanical ventilation on a patient. Let *f*_*t*_ be the clean flow signal. AutoPEEP can be regarded as the non-return of the flow signal at the end of each expiratory phase to the null value. In practice, during the observation of the air flow, various factors might get involved, including the mechanical vibration of the air tube, the patient movement, the electro-magnetic interference, etc. Therefore, the flow signal at the end of the expiratory phase will never be exactly zero, even in absence of noise. Testing directly the hypothesis $f_{t_{k}}\neq 0$, where *t*_*k*_ is the end-expiration instant of the considered breath, might thus not be realistic. A tolerance *τ *> 0 is then introduced to take into account possible distortions on the signal under consideration. Given *τ*, the problem is then the testing of $f_{t_{k}}\leq \tau$ versus $f_{t_{k}}>\tau$ based on the flow signal observation in presence of noise. This tolerance *τ* is specified by the clinician. Its value is usually derived from his/her expertise of the domain. Other technical factors could also be taken into account, such as: the flow sensor precision, the dynamic range of the signal, etc. Multiple values of *τ* could also be employed to provide a semi-quantitative evaluation of persisted AutoPEEP on patient.

**Figure 2:**
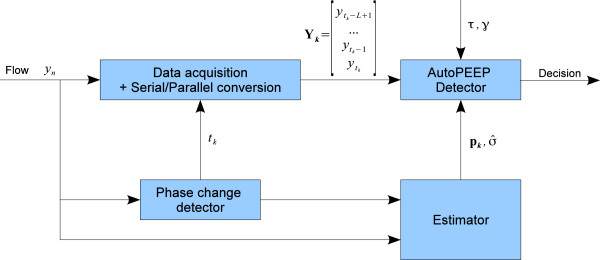
**Automatic AutoPEEP Detection Platform - System overview.** The platform functions on the basis of respiratory flow signal. For each end-expiration *t*_*k*_ it detects, the Phase change detector triggers the data acquisition/conversion process. Based on observations **Y**_*k*_ provided by the Data acquisition/conversion module and parameters $p_{k},\hat{\sigma }$ given by the Estimator, the AutoPEEP Detector performs an optimal testing with respect to specified tolerance *τ* and level *γ* to decide whether or not an AutoPEEP is present.

With respect to the discussion above, a platform for automatic detection of AutoPEEP based on a noisy observation of the flow signal can be developed. Figure 2 depicts such a platform. The main processing components include: the data acquisition and Serial/Parallel conversion, the phase-change detector, the estimator and the AutoPEEP detector. These components are briefly presented as follows before being detailed in the sequel.

### 

#### 

##### Data acquisition and Serial/Parallel conversion

This very-first module acquires the discrete flow signal *y*_*n*_ provided by the ventilator or by an independent flow sensor installed inside the air-tube during the mechanical ventilation. Although every flow datum is acquired, only end-expiration flow data of each breath is useful for the detection of AutoPEEP. When the end-expiration instant *t*_*k*_ of the *k*-th breath is provided by the phase change detector, the Data Acquisition and Serial/Parallel conversion module will log *L* samples at the end of the expiratory phase to form the observation vector $Y_{k}={\left[y_{t_{k}-L+1},y_{t_{k}-L+2},\ldots ,y_{t_{k}}\right]}^{T}$ for the *k*-th breath. This output observation vector **Y**_*k*_ is finally injected into the AutoPEEP detector module.

##### Respiration phase change detection

The main role of this module is to detect the end-expiration of each breath and provide this instant to trigger the data logging process and the Serial/Parallel conversion described above. This can also be regarded as a breath detector, which separates the continuous flow signal into different breaths.

##### Estimator

This module consists of two estimators, which estimate necessary parameters for the AutoPEEP detection algorithms. These parameters are the so-called waveform vector (**p**_**k **_for the *k*-th breath) and the noise standard deviation estimate ( $\hat{\sigma }$). The waveform vector will be used to aggregate multi-samples at the end of the expiratory phase of a breath into a decision (cf. Section Single-breath detector), while the noise standard deviation estimate will be provided to adjust the AutoPEEP detector.

##### AutoPEEP detector

The AutoPEEP detector is the main core of the whole platform. Given a specified tolerance *τ* and the desired maximum false-alarm rate (level) *γ*, the AutoPEEP detector will decide whether an AutoPEEP is present or not for a given breath, on the basis of its observation **Y**_*k*_ and estimated parameters $p_{k},\hat{\sigma }$.

### AutoPEEP detectors

Given tolerance *τ* and observation *y*_*n*_ of the noisy flow signal, the AutoPEEP detection is the testing of the null hypothesis $|f_{t_{k}}|\leq \tau$ against the alternative one $|f_{t_{k}}|>\tau$. The SNT (Signal Norm Testing) problem introduced in [7] provides such a test. In this section, two AutoPEEP detectors are proposed. One is based directly on SNT and takes each of the breaths into account independently. The other one is an extension of SNT in a sequential framework. The latter detector is developed under the assumption that the state (AutoPEEP/NON-AutoPEEP) of the patient-ventilator interface is regular and remains the same within a certain number of breaths. This assumption usually holds in practice.

#### Single-breath detector

##### Signal Norm Testing

To begin with, let us consider the signal model:

z=θ+x

where *θ* is some unknown clean deterministic signal and *z* is its observation in noise. The additive noise *x* is assumed to be centered and gaussian with variance $\sigma_{\;x}^{2}$, i.e. $x∼N(0,\sigma_{\;x}^{2})$. Given observation *z*, SNT is the problem of testing the composite hypothesis *h*_0_:|*θ*|≤*τ* versus its alternative *h*_1_:|*θ*|>*τ*.

In the sequel, a *testT* is any measurable map of ℝ into {0,1}. The value returned by *T* indicates the index of the accepted hypothesis. As in [8], the *power function* of test *T* is defined as the probability that *T* rejects the null hypothesis *h*_0_, regardless of which hypothesis actually holds, i.e.

(1) $$\beta_{\theta }(T)=P[T(z)=1].$$

The *size* of  for testing *h*_0_:|*θ*|≤*τ* is defined as the least upper bound for the probability of false-alarm, i.e.

(2) $$\alpha (T)=\underset{|\theta |\leq \tau }{\text{sup}}\beta_{\theta }(T)$$

and its *power* is the value of $\beta_{\theta }(T)$ for *θ* such that |*θ*| >* τ* — in other words, the detection probability. In practice, it is expected to maximize the power of  for a given *θ* while restricting the false-alarm rate below some level *γ*(0 <* γ *< 1). This value *γ* is specified by the clinician with respect to the acceptable number of false-alarms during a period of time. For instance, a typical value of *γ *= 0.01 corresponds to an average of one false-alarm per 5 minutes with the usual frequency of 20 [breaths/min]. The UMP (Uniformly Most Powerful) test for the problem does not exist (cf. [8]). However, the problem is invariant to any sign change in *θ*. Therefore, it is natural that the test itself should also be invariant to sign changes — that is,  should be an even function. It follows from [7] that the UMP test among those even tests with size *γ* is:

(3) $$T_{\sigma_{\;x}\lambda_{\gamma }\left(\frac{\tau }{\sigma_{\;x}}\right)}(z)=\left\{\begin{array}{lll} 1 & \;\;\text{if}\;\; & |z|\geq \sigma_{\;x}\lambda_{\gamma }\left(\frac{\tau }{\sigma_{\;x}}\right)\\ 0 & \;\;\text{if}\;\; & |z|<\sigma_{\;x}\lambda_{\gamma }\left(\frac{\tau }{\sigma_{\;x}}\right) \end{array}\right)$$

in which *λ*_*γ*_(*ρ*) is the unique solution in *η* to the equation 1−*Φ*(*η*−*ρ*)−*Φ*(−*η*−*ρ*)]=*γ*, where *Φ*(.) is the cumulative distribution of any standard normal random variable. Additionally, the test is UMPU (UMP unbiased) [7]. This thresholding test will be used for the detection of AutoPEEP, one of the most frequent abnormalities exhibited during mechanical ventilation.

#### Single-breath SNT-based AutoPEEP detector

Although the definition of AutoPEEP is based solely on the final sample of the expiratory phase of each breath, it is expected that taking multiple samples into account will improve the detection performance. By introducing the waveform vector, namely **p**_*k*_, with dimension *L*, one can aggregate *L* samples at the end of the expiration to carry out a single decision for the breath under consideration. Let **Y**_*k*_ be the observation vector containing the last *L* samples of the expiratory phase of the *k*-th breath under consideration. **Y**_*k*_ is modeled as:

$$Y_{k}=f_{k}+X_{k}$$

where $f_{k}={\left[\;f_{t_{k}-L+1}\;\;\ldots \;\;\;f_{t_{k}-1}\;\;\;f_{t_{k}}\right]}^{T}$ is the flow signal vector and $X_{k}∼N(0,\sigma^{2}I_{L})$ is additive gaussian noise with standard deviation *σ*. Vector **f**_*k*_ can be factorized as:

$$f_{k}=p_{k}f_{t_{k}}$$

where $p_{k}={\left[p_{1}^{\left(k\right)}\;\;\;p_{2}^{\left(k\right)}\;\;\;\ldots \;\;\;p_{L}^{\left(k\right)}\right]}^{T}$ is the waveform vector. It should be noted that $p_{L}^{\left(k\right)}=1$. This vector **p**_*k*_ corresponds to the local form of the flow signal near the end of the expiratory phase. It is also worth mentioning that this local waveform vector **p**_*k*_ depends mainly on the configuration of the interface, including the patient condition and the ventilator settings. As long as the interface stays unchanged, the waveform vector remains almost the same regardless whether or not an AutoPEEP might occur. In practice, either **p**_*k*_ is known prior to the detection or it can be estimated from the observation using one of the methods proposed in Section Waveform regression to compute **p**_*k*_.

To aggregate *L* observed samples into one decision for the considered breath, **Y**_*k*_ is projected onto the direction generated by **p**_*k*_. We thus have:

(4) $$u_{k}=f_{t_{k}}+w_{k}$$

where $u_{k}=p_{k}^{T}Y_{k}/∥p_{k}{∥}^{2},\;w_{k}=p_{k}^{T}X_{k}/∥p_{k}{∥}^{2}$ and $∥p_{k}{∥}^{2}=p_{k}^{T}p_{k}$ is the *L*_2_ norm of waveform vector **p**_*k*_. By such proceeding, noise *w*_*k*_ follows normal distribution with zero mean and variance $\sigma_{w}^{2}=\sigma^{2}/∥p_{k}{∥}^{2}$. According to [9, Theorem 27.4, p. 362] and equation (14), it can be proved that, even when the original noise is not gaussian, the resulting noise *w*_*k*_ tends to a normally distributed random variable, as long as *L* is large enough and the original noise samples are i.i.d (independent and identically distributed). In practice, the i.i.d condition can be significantly relaxed. The problem in (4) is the same as that in previous section, except that the noise level is reduced (*σ*_*w *_≤* σ*). Moreover, no information on the correlation among samples of noise vector **X**_*k*_ is required. The two hypotheses are unchanged: $h_{0}:|f_{t_{k}}|\leq \tau$ and $h_{1}:|f_{t_{k}}|>\tau$. The detection is thus carried out as follows. We decide that there is an AutoPEEP if $\left|u_{k}|>\sigma_{w}\lambda_{\gamma }(\frac{\tau }{\sigma_{w}}\right)$, where *λ*_*γ*_(.) is calculated as in (3). Otherwise, the considered breath is labeled with Non-AutoPEEP.

It should be noted that ∥**p**_*k*_∥ increases with respect to the number *L* of samples. The noise standard deviation *σ*_*w*_ will thus decreases when more samples are taken into account. By reducing the noise standard deviation, the detection probability is improved while the false-alarm rate is still limited to the specified level *γ*. Theoretically, *L* is only limited by the length of expiratory phase. However, *L* must not be too long so that the local waveform vector can be considered stable and stays almost unchanged for a large number of breaths.

#### Sequential detector

##### SNT extension in sequential decision framework

By using SNT, one can restrict the false-alarm rate to some value *γ*. It is has also been shown in [7] that the detection rate is lower-bounded by *γ*. Since *γ* is usually be very small, this bound is of poor interest. In this section, an extension of SNT in a sequential decision framework — namely, the *Sequential SNT* — is introduced to improve the detection rate while still limiting the false-alarm rate to the specified value *γ*. The main idea is to introduce two detection thresholds: one is calculated to restrict the false-alarm rate as described in Section Single-breath detector, whereas the other is obtained by exchanging the two hypotheses to limit the miss-detection rate. In particular, with the same notation as in Section Single-breath detector, we take both the from-above testing problem considered in Section Single-breath detector:

(5) $$\left\{\begin{array}{ll} h_{0}: & \;\;|\theta |\leq \tau \\ h_{1}: & \;\;|\theta |>\tau \end{array}\right)$$

and the from-below one:

(6) $$\left\{\begin{array}{ll} h_{0}^{\prime }: & \;\;|\theta |>\tau \\ h_{1}^{\prime }: & \;\;|\theta |\leq \tau \end{array}\right)$$

into account. On the one hand, put $\lambda_{\gamma }^{*}=\sigma_{\;x}\lambda_{\gamma }(\frac{\tau }{\sigma_{\;x}})$. As aforementioned, the test:

$$T_{\lambda_{\gamma }^{*}}(z)=\left\{\begin{array}{lll} 1 & \;\;\text{if}\;\; & |z|>\lambda_{\gamma }^{*}\\ 0 & \;\;\text{if}\;\; & |z|\leq \lambda_{\gamma }^{*} \end{array}\right)$$

has size *γ* for problem (5) so that

(7) $$P\left[|z|>\lambda_{\gamma }^{*}\right]\leq \gamma \;\;\text{when}\;\;|\theta |\leq \mathrm{\tau .}$$

$T_{\lambda_{\gamma }^{*}}$ is called the thresholding test from above with threshold height $\lambda_{\gamma }^{*}$. On the other hand, according to [7], the UMP (Uniformly Most Powerful) test with size *γ* for problem (6) also exists and is the thresholding test from below with threshold height $\lambda_{1-\gamma }^{*}=\sigma_{\;x}\lambda_{1-\gamma }(\frac{\tau }{\sigma_{\;x}})$:

$$T_{\lambda_{1-\gamma }^{*}}(z)=\left\{\begin{array}{lll} 1 & \mathrm{if} & |z|\leq \lambda_{1-\gamma }^{*}\\ 0 & \mathrm{if} & |z|>\lambda_{1-\gamma }^{*} \end{array}\right)$$

We also have:

(8) $$P\left[|z|<\lambda_{1-\gamma }^{*}\right]\leq \gamma \;\;\text{when}\;\;|\theta |>\tau .$$

For *γ* <0.5, it follows from [7] that $\lambda_{1-\gamma }^{*}<\lambda_{\gamma }^{*}$. It is also worth mentioning that *γ* is usually set to be small. In practice, *γ*=0.001,0.005,0.01,0.05 are typical values. Using both $\lambda_{\gamma }^{*}$ and $\lambda_{1-\gamma }^{*}$, let us consider the test:

(9) $$T^{*}(z)=\left\{\begin{array}{ll} 1 & \text{if}\;\;|z|>\lambda_{\gamma }^{*}\\ 0 & \text{if}\;\;|z|\leq \lambda_{1-\gamma }^{*}\\ ?(\text{not decided yet}) & \text{otherwise} \end{array}\right)$$

for testing the hypothesis *h*_0_:|*θ*| ≤* τ* against the alternative one *h*_1_:|*θ*| >* τ*. It is inherited from (7) and (8) that, once the decision has been made by (9), the probability of false-alarm (*P*_FA_) is limited to the specified value *γ* (i.e. *P*_FA_ <* γ*) and the detection probability (*P*_D_) is guaranteed to be higher than 1−*γ*(i.e. *P*_D_ > 1 −*γ*).

In a sequential framework, the test $T^{*}(z)$ is firstly carried out to attempt a decision based on current observation *z*. If $T^{*}(z)$ returns 1 or 0 then the decision is made. The value returned by $T^{*}(z)$ is the index of the accepted hypothesis. Otherwise, the decision cannot be made yet since current observation *z* does not provide enough evidence to either accept or reject any of the two hypotheses. More data are required. The decision is then postponed until enough evidence has been collected. For AutoPEEP detection, the process is detailed below.

#### Sequential SNT-based AutoPEEP detector

Let us consider *K* consecutive breaths. Using the same aggregation scheme as in Section Single-breath detector for each breath, we have:

(10) $$u_{k}=f_{t_{k}}+w_{k}\;\;\text{for}\;\;k=1,2,\ldots ,K$$

and $w_{k}\overset{\mathrm{iid}}{∼}N(0,\sigma_{w}^{2})$. By averaging over the *K* breaths, we obtain:

(11) $$u_{1:K}=f_{t_{1:K}}+w_{1:K}$$

where:

$$\begin{array}{lll} u_{1:K}=\frac{1}{K}\overset{K}{\underset{k=1}{\sum }}u_{k}, & \;f_{t_{1:K}}=\frac{1}{K}\overset{K}{\underset{k=1}{\sum }}f_{t_{k}}, & \;w_{1:K}=\frac{1}{K}\overset{K}{\underset{k=1}{\sum }}w_{k}. \end{array}$$

It is worth mentioning that $w_{1:K}∼N(0,\sigma_{w,K}^{2})$ and that $\sigma_{w,K}=\frac{\sigma_{w}}{\sqrt{K}}$ is strictly decreasing with the number *K* of breaths used.

Assuming that the true hypothesis (AutoPEEP/NON-AutoPEEP) remains the same for *K* consecutive breaths, the AutoPEEP detection for these breaths amounts to determining whether or not the average end-expiration flow $f_{t_{1:K}}$ exceeds the specified tolerance *τ*. Given level *γ*, the from-above test for this problem is:

$$T_{\lambda_{1:K}^{\left(h\right)}}(u_{1:K})=\left\{\begin{array}{lcc} 1 & \text{if} & |u_{1:K}|>\lambda_{1:K}^{\left(h\right)}\\ 0 & \text{if} & |u_{1:K}|\leq \lambda_{1:K}^{\left(h\right)} \end{array}\right)$$

for testing $h_{0}:|f_{t_{1:K}}|\leq \tau$ against $h_{1}:|f_{t_{1:K}}|>\tau$ and the from-below test is:

$$T_{\lambda_{1:K}^{\left(\ell \right)}}(u_{1:K})=\left\{\begin{array}{lcc} 0 & \text{if} & |u_{1:K}|>\lambda_{1:K}^{\left(\ell \right)}\\ 1 & \text{if} & |u_{1:K}|\leq \lambda_{1:K}^{\left(\ell \right)} \end{array}\right)$$

for testing $h_{0}^{\prime }:|f_{t_{1:K}}|>\tau$ against $h_{1}^{\prime }:|f_{t_{1:K}}|\leq \tau$. The two associated thresholds are thus:

$$\lambda_{1:K}^{\left(h\right)}=\sigma_{w,K}\lambda_{\gamma }(\tau /\sigma_{w,K})$$

and

$$\lambda_{1:K}^{\left(\ell \right)}=\sigma_{w,K}\lambda_{1-\gamma }(\tau /\sigma_{w,K})$$

where $\lambda_{1:K}^{\left(h\right)}>\lambda_{1:K}^{\left(\ell \right)}$ for any 0<*γ*<0.5.

Summarizing, in a sequential decision framework, the AutoPEEP detection is carried out as follows. Firstly, the detector tries to make a decision based solely on the observation of the first breath, using the test:

$$T^{*}(u_{1:1})=\left\{\begin{array}{lcc} 1 & \text{(AutoPEEP)} & \text{if}\;\;|u_{1:1}|>\lambda_{1:1}^{\left(h\right)}\\ 0 & \text{(NON-AutoPEEP)} & \text{if}\;\;|u_{1:1}|\leq \lambda_{1:1}^{\left(\ell \right)}\\ ? & \text{(not decided yet)} & \text{otherwise} \end{array}\right)$$

If the decision cannot be made yet (i.e. $\lambda_{1:1}^{\left(\ell \right)}\leq |u_{1:1}|\leq \lambda_{1:1}^{\left(h\right)}$), it will be delayed until the next observation (i.e. *u*_2_) is obtained and the test is performed based on *u*_1:2_ using $T^{*}(u_{1:2})$. If the decision still cannot be performed, it will be delayed again until the next observation, where the test $T^{*}(u_{1:3})$ is used. The process is iterated until the decision is made. Then the process is restarted for a new sequence of observations.

**Figure 3:**
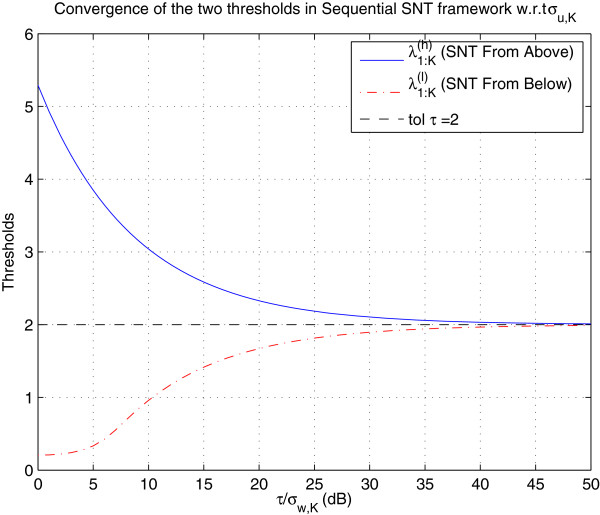
**Thresholds convergence.** This figure illustrates the convergence of the two thresholds in Sequential SNT framework. This convergence suggests that, in sequential SNT framework, the decision will probably be made after a finite number of samples are acquired.

As shown in Figure 3, both $\lambda_{1:K}^{\left(h\right)}$ and $\lambda_{1:K}^{\left(\ell \right)}$ tend to tolerance *τ* when $\sigma_{w,K}\to 0$ (the proof will be carried out in a future work which covers all theoretical aspects of Sequential SNT). It should also be noted that $\sigma_{w,K}\underset{K\to \infty }{\to }0$. Therefore, it could be expected that the probability to make a decision is one when K→∞. However, if *K* is too high, the assumption that the same state remains for the considered *K* consecutive breaths might no longer hold. Moreover, a high number of breaths to be acquired may yield an unacceptable delay-to-decision. One simple solution is then to limit the number of breaths to some value *M*. If *M* breaths have been observed but no decision has been made, a *hard* decision is then performed. To assure the false-alarm rate, threshold $\lambda_{1:M}^{\left(h\right)}$ is used. The hard decision is carried out by:

$$T_{\lambda_{1:M}^{\left(h\right)}}(u_{1:M})=\left\{\begin{array}{lcc} 1 & \text{(AutoPEEP)} & \text{if}\;|u_{1:M}|\;>\;\lambda_{1:M}^{\left(h\right)}\\ 0 & \text{(NON-AutoPEEP)} & \text{if}\;|u_{1:M}|\;\leq \;\lambda_{1:M}^{\left(h\right)} \end{array}\right)$$

The value *M* must be chosen so that the assumption mentioned above holds valid and the delay-to-decision is still in an acceptable range. In our experimental settings, we use *M*=10, which corresponds to about 30 seconds of observation in the usual case with a breathing frequency of 20 [breaths/min].

### Phase change detection

Since the detection is performed on the basis of the flow samples at the end of the expiratory phase of each breath, it is required that the end-expirations are precisely retrieved. As aforementioned, the main role of the Phase change detection/segmentation block is to provide a detection of the end-expiration for each breath. This can be achieved by detecting the change in flow signal *y*_*n*_ from the expiratory phase of the current breath (negative values) to the inspiratory phase of the next breath (positive values) (c.f. Figure 1). As long as the distortion of the signal caused by noise gets involved and may bias the detection, a smoothed version of the signal can be used. To be simple, the *moving average smoothing* (SMA) method can be considered:

$${\bar{y}}_{n}=\text{SMA}(y_{n})=\frac{1}{2h+1}\overset{i=n+h}{\underset{i=n-h}{\sum }}y_{i}$$

where 2*h* + 1 is the length of the moving window.

**Figure 4:**
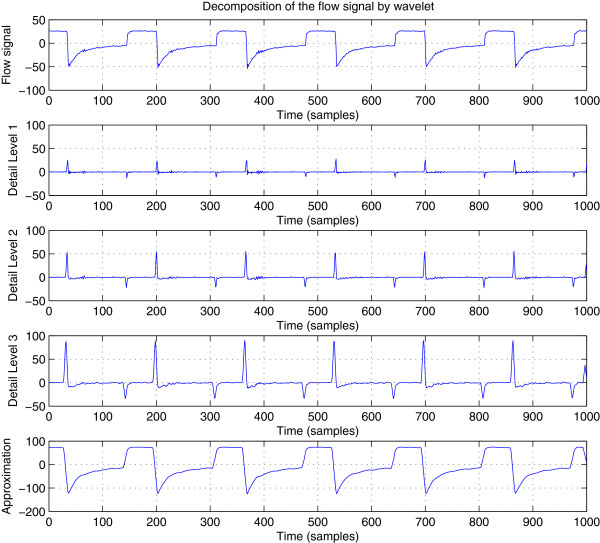
**Wavelet decomposition of the flow signal.** The peaks in detail bands correspond to changes from inspiratory phase to respiratory phase and vice versa.

Since the wavelet transform is a powerful processing tool to retrieve irregularities in a signal, it can be used to carry out the detection of change from the expiratory phase of a breath to the inspiratory phase of the next one. The wavelet transform is applied on either the flow signal *y*_*n*_ on its smoothed version ${\bar{y}}_{n}$. End-expirations are actually negative peaks in the detail coefficients (high band). Figure 4 shows an example of these peaks. In this example, the discrete stationary wavelet transform was used and the number of wavelet decomposition levels was set to *K *= 3.

The end-expiration detection is performed by thresholding these peaks in the detail bands of the wavelet transform coefficients. Let us consider the level-2 detail band for instance. This detail band signal is composed of noise and peaks, which represent the irregularities in the original flow signal. Since the flow signal is supposed to be in independent additive gaussian noise and since the wavelet transform is linear, the noise in detail band is also gaussian. Therefore, each coefficient in the detail band can be modeled as *y*_*D *_=* f*_*D*_ + *x*_*D*_, where *f*_*D*_ is a signal coefficient and *x*_*D*_ is gaussian noise. Let *σ*_*D*_ be the standard deviation of this noise and let *N* be the number of coefficients. It was shown in [10-12] that the universal threshold: $\lambda_{u}(N)=\sigma_{D}\sqrt{2\ln N}$ can be interpreted as the noise maximum absolute value when *N* is large enough. This threshold *λ*_*u*_(*N*) can also be thought of as the minimum absolute value of the signal (cf. [13]). Therefore, the problem amounts to testing the peak absolute value with respect to *λ*_*u*_(*N*). This is, once again, a SNT problem in the sense given by [7] and Section Single-breath detector. The peaks in the detail band can thus be detected using the test:

$$T_{\lambda_{\text{SNT}}}(y_{D})=\left\{\begin{array}{lcc} 1 & \text{if} & |y_{D}|>\lambda_{\text{SNT}}\\ 0 & \text{if} & |y_{D}|\leq \lambda_{\text{SNT}} \end{array}\right)$$

with threshold height $\lambda_{\text{SNT}}=\sigma_{D}\lambda_{\gamma }\left(\lambda_{u}(N)/\sigma_{D}\right)$ where *λ*_*γ*_(*ρ*) is defined as in Section Single-breath detector. Level *γ* is set to be very small, for example *γ *= 10^−4^,10^−5^,10^−7^, etc. Since a peak is only one point, the results of the thresholding test should be post-processed in such a way that consecutive 1s are removed. In particular, in case of consecutive decisions equal to 1, only the first one will be kept. End-expirations are negative peaks.

**Figure 5:**
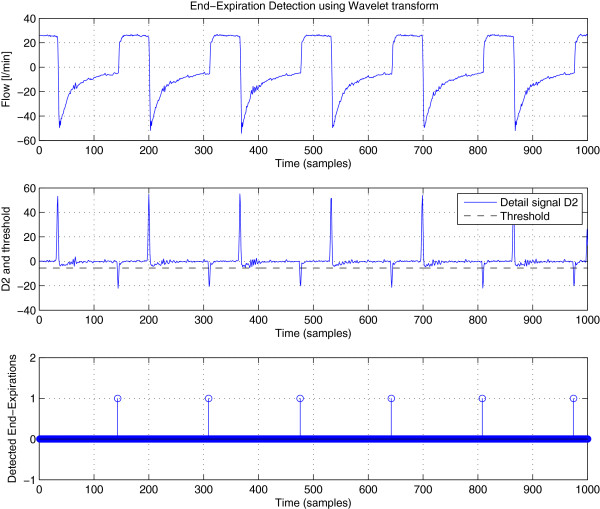
**End-Expiration Detection using Wavelet transform.** This figure illustrates the detection of end-expirations based on respiratory flow signal: (top) respiratory flow curve obtained from a patient, (middle) signal in the level-2 detail band of the wavelet transform coefficients and the calculated detection threshold, (bottom) detection result, where 1s (peaks) represent end-expirations.

As long as noise standard deviation *σ*_*D*_ is concerned, it can be estimated using the same methods as those described in Section Estimation of the noise standard deviation. Figure 5 gives a typical result of the end-expiration detection obtained by proceeding as described above.

### Estimation

As aforementioned, some estimations have to be made prior to the AutoPEEP detection, including: the waveform vector (**p**_*k*_) and the standard deviation of the unknown noise. In the following, these two estimations will be addressed.

#### Waveform regression to compute **p**_*k*_

**Figure 6:**
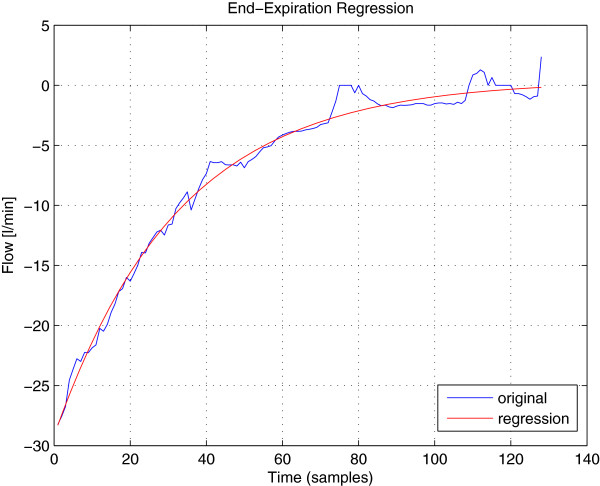
**Fitness of the model function.** An example of the flow signal at the end of an expiratory phase with its regression curve using the model function in (12). The result firmly shows the relevance of the considered model function to the regression task.

With regard to Section Single-breath detector, the waveform vector **p**_*k*_ is the key which makes it possible to aggregate multiple end-expiration flow samples into one decision. This vector **p**_*k*_ can be calculated from the regression of the flow signal at the end of the expiration. Indeed, during the expiratory phase of a breath, the mechanical ventilation system works based solely on the passive response of the patient lung. Due to the resistance of the airways and the elasticity of human lung, the flow signal during the expiratory phase of a breath can be modeled by:

(12) $$y(t)=C-\phi e^{-\mathrm{\mu t}},$$

with *ϕ *> 0 and *μ *> 0, even in presence of AutoPEEP. This model is used to estimate the referenced waveform at the end of the expiration using a nonlinear robust regression method. Given a set of *N* data points {(*t*_*i*,_*y*(*t*_*i*_)),*i *= 1*..N*} where *y*(*t*_*i*_) is the observation at instant *t*_*i*_, the non-linear robust regression aims at solving the least square problem:

(13) $${\left(C,\phi ,\mu \right)}^{*}=\underset{C,\phi ,\mu }{\arg\min}\overset{N}{\underset{i=1}{\sum }}\xi_{i}{\left[y(t_{i})-(C-\phi e^{-\mu t_{i}})\right]}^{2}$$

where the introduction of weight vector [*ξ*_1_,*ξ*_2_,*..ξ*_*N*_] makes it possible to reduce the influence of outliers onto the final result. The MATLAB routine *nlinfit* performs such a regression task. This routine uses a weighted version of the Levenberg-Marquardt algorithm [14] to solve the non-linear least squares problem (13). The weights are iteratively updated with respect to corresponding residues $|y(t_{i})-(C-\phi e^{-\mu t_{i}})|,i=1\mathrm{..n}$ to downweight the outliers and therefore reduce their effects on the final regression curve. Figure 6 shows an example of the flow signal at the end of the expiratory phase and the regression resulting from the aforementioned non-linear robust method. The signal is shown to be well-fitted by the model function (12).

**Figure 7:**
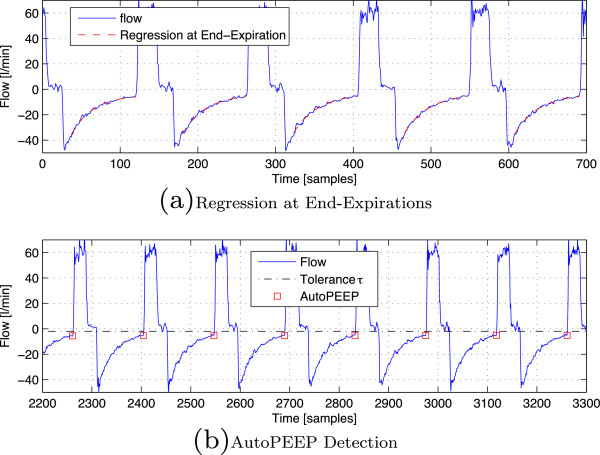
Detection results on clinical data.

Even though only *L* samples are required to calculate the *L*-dimensional vector **p**_*k*_, more samples should be used to achieve a better regression curve. Let *L*_ext_(*L*_ext_ ≥* L*) be the number of samples to be used. *L*_ext_ is only limited by the length, namely *T*_*e*_ (in samples), of the expiratory phase, i.e. *L*_ext_ ≤* T*_*e*_. Regarding the transition between different respiratory phases, samples at the beginning of the expiration are very sensitive to transition and may bias the regression. Therefore, only a proportion of the *T*_*e*_ samples of the expiratory phase should be taken into account:

$$L_{\text{ext}}=\left\lfloor \alpha T_{e}\right\rfloor$$

where 0 <* α *< 1 is the proportion and ⌊.⌋ is the *floor* function. Proportion *α* must be chosen so that $L_{\text{ext}}=\left\lfloor \alpha T_{e}\right\rfloor \geq L$. Additionally, to avoid the border effect, one might consider the weighted regression with a weighting scheme that puts more weight on the middle samples than on the side ones. Figure 7a shows the regression on end-expiration samples of a flow signal recorded from a patient. In this example, *α*is set to 0.75 to avoid the transition effects at the beginning of the expiratory phase. Since it is not so crucial in the situation experienced in this work, an unweighted regression was employed.

Given the regression at the end of expiration, namely $\left[{ŷ}_{t_{k}-L_{\text{ext}}+1},{ŷ}_{t_{k}-L_{\text{ext}}+2},\ldots ,{ŷ}_{t_{k}}\right]$, the last *L* values are used to calculate the estimate ${\hat{p}}_{k}$ for the considered breath:

(14) $${\hat{p}}_{k}={\left[{ŷ}_{t_{k}-L+1},{ŷ}_{t_{k}-L+2},\ldots ,{ŷ}_{t_{k}}\right]}^{T}/{ŷ}_{t_{k}}$$

According to Section Single-breath detector, waveform vector **p**_*k*_ concerns the current (*k*-th) breath. However, as aforementioned, this waveform vector does not vary much. It is then sensible to use estimates from previous breaths so as to improve the estimation of **p**_*k*_. In this respect, the following strategies can be considered to compute the waveform vector estimate to be used in AutoPEEP detectors: 

"**Static waveform vector:** The waveform vector is computed based on the first *N*_ref_ breaths right after a verification/tuning session of the clinician. These *N*_ref_ breaths are used as reference after validation by the clinician.

$$p_{k}=\frac{1}{N_{\text{ref}}}\overset{N_{\text{ref}}}{\underset{k=1}{\sum }}{\hat{p}}_{k}$$

This waveform vector will be updated each time the machine is tuned or after a verification session by the clinician. One may also want to update the estimation on a regular time basis.

**Dynamic waveform vector:** The waveform vector to be used is the one estimated from the current breath:

$$p_{k}={\hat{p}}_{k}$$

**Adaptive waveform vector:** In this strategy, the waveform vector is updated every time a new breath is observed. Previous estimates are taken into account with a forgetting factor *μ* such that 0 <* μ*< 1:

$$p_{k}=\frac{1-\mu }{1-\mu^{k}}\overset{k}{\underset{i=1}{\sum }}\mu^{k-i}{\hat{p}}_{i}$$

#### Estimation of the noise standard deviation

Noise is unknown in practice. As long as the noise standard deviation in concerned, it must be estimated from the observation. In this work, we propose two solutions: one based directly on the result obtained by waveform regression, whereas the other is based on an estimation from the wavelet coefficients of the flow signal.

#### Estimate from regression

By using the regression, the residue can be considered as noise. Therefore, the noise standard deviation can be estimated directly from this residue. For the *k*-th breath, we have:

$${\hat{\sigma }}_{k}=\frac{1}{L_{\text{ext}}-1}\sqrt{\overset{t_{k}}{\underset{i=t_{k}-L_{\text{ext}}+1}{\sum }}{\left(y_{i}-{ŷ}_{i}\right)}^{2}}$$

To aggregate $\hat{\sigma }$ from ${\hat{\sigma }}_{k}$, the same strategies as those proposed for the waveform vector can be considered.

#### Estimation from wavelet coefficients

Studies on nonparametric estimation based on Wavelet Shrinkage have shown that most of the wavelet coefficients obtained from the first level wavelet decomposition of a piecewise smooth signal are of very small amplitude. Only a small number of these wavelet coefficients, which correspond to signal, are of higher amplitude [12]. This fact allows the use of robust estimators on the wavelet coefficients to provide noise estimation. One can consider the MAD (median absolute deviation) [15,16] to accomplish such a task. The method is usual [12,15,16] and we recall it for readiness sake. Let *c*_1_,*c*_2_,…*c*_*N*_ be the wavelet coefficients obtained from the first level discrete wavelet decomposition of an *N*-sample segment of the flow signal *y*. The estimate ${\hat{\sigma }}_{\text{MAD}}$ of *σ* is then provided by:

$${\hat{\sigma }}_{\text{MAD}}=b\times {\text{med}}_{i}|c_{i}-{\text{med}}_{j}c_{j}|$$

where *b*≈1.4826. Since the noise is central, white and gaussian, the formula is simplified to:

$${\hat{\sigma }}_{\text{MAD}}=b\times {\text{med}}_{i}|c_{i}|$$

knowing that med_*i*_*c*_*i *_= 0.

In [17], another robust estimator was proposed, namely the *d-dimensional adaptive trimming estimator* (DATE). The method is summarized as follows. Let *c*_(1)_*c*_(2)_,…,*c*_(*N*)_ be sequence of wavelet coefficients *c*_1_,*c*_2_,…*c*_*N*_ sorted by increasing magnitude. Put $m_{\text{min}}=\frac{N}{2}-\sqrt{\frac{N}{4(1-Q)}}$ where *Q *= 0.95. Let *m* be the smallest integer, *m*_min_≤*m*≤*N* such that:

$$\left|c_{\left(m\right)}|\leq 2.7238\times \frac{1}{m}\overset{m}{\underset{k=1}{\sum }}|c_{\left(k\right)}|<|c_{\left(m+1\right)}\right|$$

If such an integer *m* does not exist, set *m *=* m*_min_. The estimate ${\hat{\sigma }}_{\text{DATE}}$ of *σ* is then provided by:

$${\hat{\sigma }}_{\text{DATE}}=1.2533\times \frac{1}{m}\overset{m}{\underset{k=1}{\sum }}|c_{\left(k\right)}|$$

It has been shown in [17] that this estimator outperforms the MAD when the number of outliers increases. The DATE can thus be employed as an alternative to the MAD mentioned above in such situation. For the cases considered in this work, because the number of large wavelet coefficients pertaining to signal remains small, the two estimators yield similar performance. The MAD estimator is thus adopted for its lower complexity and higher rapidity.

## Results and discussion

### Simulations

**Figure 8:**
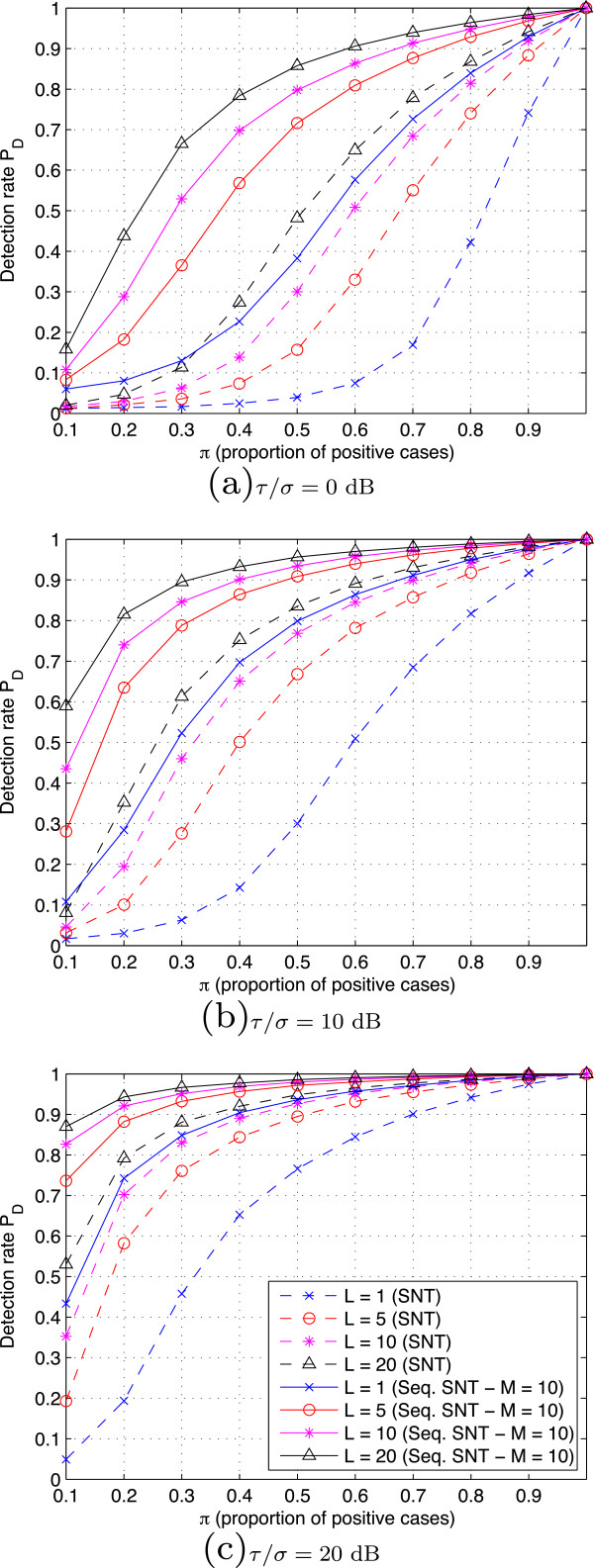
**Detection curves yielded by the two proposed AutoPEEP detectors with different noise levels.** The simulations were carried out with *N*=10000 breaths, tolerance *τ*=2 [l/min] and level *γ*=0.01. With the extension of SNT in a sequential framework, the resulting detector yields a significant improvement in detection rate while the false alarm is still limited to the specific value *γ*.

To illustrate the detection performance of the proposed algorithms, the flow signal was first synthesized on computer. For each breath, *L* end-expiration samples were generated. The waveform vector was supposed to be known and set to $p_{k}=p={\left[1,1,\ldots ,1\right]}^{T}$. It is worth mentioning that, by construction, |*p*_1_|≥|*p*_2_|≥…≥|*p*_*L*_|=1 and, as a result, $\sigma_{w}=\frac{\sigma }{∥p_{k}∥}\leq \frac{\sigma }{\sqrt{L}}$. The equality happens when and only when *p*_*i*_=1 for all *i*=1*..L*. With regard to noise level *σ*_*w*_, by setting $p_{k}=p={\left[1,1,\ldots ,1\right]}^{T}$, we considered the worst case where $∥p_{k}{∥}^{2}=L$ and $\sigma_{w}=\frac{\sigma }{\sqrt{L}}$. For sequential SNT-based detector, *M* was set to 10 [breaths], which corresponds to about 30 seconds of observation. The tolerance was empirically set to *τ*=2 [l/min] by clinician. The values of $f_{t_{k}}$ were randomly and uniformly generated between 0 and $-\frac{\tau }{1-\Pi }$, where *Π*is the proportion of positive cases (AutoPEEP). Since the false-alarm rate *P*_FA_ is always restricted to the specified value *γ*, it is more meaningful to plot the detection rate *P*_D_ versus different values of *Π*, namely the *detection curve*, than to present the usual ROC (Receiver Operating Curve). Figure 8 shows detection curves for different noise levels and different values of *L*. The detection rate is significantly improved when more samples are aggregated. Of course, the lower the noise level, the better the detection. In this respect, the Sequential SNT-based detector also showed higher detection rate while still keeping the false-alarm rate below the specified value *γ*.

### Emulations with a respiratory system analog

The proposed AutoPEEP detectors were also tested in a more realistic setting in which the interface between a ventilator and a lung model was established. In these experiments, the respiratory system analog was constituted by a G5 ventilator (Hamilton Medical, Bonaduz, Switzerland) connected to the ASL5000 computerized lung model (Ingmar Medical Ltd., Pittsburgh, PA, USA), making it possible to modify respiratory mechanics. Thirteen sets of parameters (cf. Table 1) for both the lung emulator and the ventilator, which correspond to various practical situations, were carried out. The tolerance *τ *= 2 [l/min] was employed again. With respect to this tolerance, among the 13 settings, 7 cases were reported as AutoPEEP and the other 6 cases were labeled as NON-AutoPEEP, thanks to an independent clinical analysis from the Intensive Care unit of Brest University Hospital, Brest, France. The detection was performed on the basis of the flow signal captured by the sensor integrated in the ASL5000 lung model. For each case, about 1.5 minute of the signal flow was recorded. The corresponding number of breaths varied from 13 to 34, depending on the setting. In total, 323 breaths were recorded. For both the proposed detectors, the dynamic waveform vector was employed for its simplicity. Level *γ* was set to 0.01. The detection results are reported in Table 1. All the 13 cases were successfully detected by the two proposed methods: the Single-breath SNT-based detector and the Sequential SNT-based detector. Moreover, in each case, all the breaths were precisely classified. No detection error was found among the 323 breaths analyzed.

**Table 1:** AutoPEEP detection results provided by the proposed detectors on emulated flow data

	**Parameters**		**True**	**N. of**	**Det. by SNT**^c^			**Det. by Sequential SNT**^c^		
**Id**	**Ventilator**^a^	**Lung model**^b^	**Label**	**breaths**	**P**	**N**	**Label**	**P**	**N**	**Label**
1	PEP=0, Vt=500, f=15, P=0, I:E=1:2	C=80, R=5	N	21	0	21	N	0	21	N
2	PEP=0, Vt=500, f=15, P=0, I:E=1:2	C=30, R=5	N	20	0	20	N	0	20	N
3	PEP=0, Vt=500, f=25, P=0, I:E=1:2	C=80, R=5	P	33	33	0	P	33	0	P
4	PEP=0, Vt=500, f=25, P=0, I:E=1:1	C=80, R=5	P	34	34	0	P	34	0	P
5	PEP=0, Vt=300, f=20, P=0, I:E=1:2	C=80, R=5	N	27	0	27	N	0	27	N
6	PEP=0, Vt=500, f=12, P=0, I:E=1:2	C=80, R=5	N	16	0	16	N	0	16	N
7	PEP=0, Vt=500, f=20, P=15, I:E=1:3	C=80, R=5	N	27	0	27	N	0	27	N
8	PEP=5, Vt=500, f=20, P=0, I:E=1:3	C=80, R=5	N	27	0	27	N	0	27	N
9	PEP=5, Vt=500, f=20, P=0, I:E=1:2	C=120, R=10	P	27	27	0	P	27	0	P
10	PEP=0, Vt=700, f=20, P=0, I:E=1:2	C=120, R=10	P	27	27	0	P	27	0	P
11	PEP=0, Vt=700, f=20, P=0, I:E=1:6	C=120, R=10	P	24	24	0	P	24	0	P
12	PEP=0, Vt=700, f=20, P=0, I:E=1:1	C=120, R=10	P	27	27	0	P	27	0	P
13	PEP=0, Vt=700, f=20, P=0, I:E=1:2	C=140, R=25	P	13	13	0	P	13	0	P

^a^Ventilator parameters include: Positive Expiratory Pressure PEP [cm*H*_2_*O*], air volume Vt [ml], frequency f [breaths/min], pause time P [%], Inspiratory to expiratory time ratio I:E.

^b^Lung model parameters include: compliance C [ml/cm*H*_2_*O*] and resistance R [cm*H*_2_*O*/l/s].

^c^For each of the experiments, the AutoPEEP detection provides: the number of breaths detected as AutoPEEP (denoted as P for Positive), the number of breaths detected as NON-AutoPEEP (denoted as N for Negative) and the overall label for the considered setting.

### Analysis of clinical data

For further evaluation, the AutoPEEP detectors were tested *ex-vivo* on various patient curves. These curves were retrospectively extracted from data files issued from the Medical Intensive Care Unit of Brest University Hospital, France and from the Institut Universitaire de Cardiologie et de Pneumologie de Québec, Canada. For each patient undergoing mechanical ventilation, the flow signal was recorded. All these data were then mixed up to form a unique dataset. In total, the final dataset contains 1998 breaths from 15 patients with different health conditions and different treatments. The parameters of the ventilator also varied depending on the situation. According to the retrospective aspect of the study and to the fact that the files were anonymized, the study was considered to be in accordance with French legislation by our local ethics committee.

The analysis was performed both manually by a set of experts and automatically by the proposed methods. On the one hand, each breath was carefully screened by two experts of the domain. They performed a dual analysis, separately, before confronting their points of view and delivering a final assessment of the data. For each breath of the dataset, their decision was then regarded as the ground-truth label (AutoPEEP/NON-AutoPEEP). On the other hand, the proposed detectors were used to predict the label of every breath of the dataset. The two analyses were carried out independently and anonymously. The results were then compared together to evaluate the detection performance of the proposed methods.

In these experiments, the tolerance was set to *τ *= 2 [l/min] as before. In this respect, the dataset includes 1383 breaths with AutoPEEP and 615 breaths with NON-AutoPEEP. The dataset is somehow unbalanced with the presence of AutoPEEP in 69% of the cases. For the proposed detectors, level *γ* was set to 0.01 as usual. Figure 7 presents a typical case with the regression at end-expiration and the corresponding detection. It can be seen that the detection algorithm can precisely reveal the true label for all the breaths.

To quantitatively assess the detection performance of the proposed methods, we considered four usual evaluation measures: Accuracy, Precision, Recall (Sensitivity) and Specificity. These measures are defined as follows:

$$\text{Accuracy}=\frac{T\;P+T\;N}{T\;P+T\;N+F\;P+F\;N}$$

$$\text{Precision}=\frac{T\;P}{T\;P+F\;P}$$

$$\text{Recall (Sensitivity)}=\frac{T\;P}{T\;P+F\;N}$$

$$\text{Specificity}=\frac{T\;N}{T\;N+F\;P}$$

where: *TP* (resp. *TN*) is the number of true positives (resp. true negatives), defined as the number of breaths with (resp. without) AutoPEEP that are correctly predicted; *FP* (false positive) is the number of breaths without AutoPEEP that are falsely predicted as AutoPEEP, and *FN*(false negative) is the number of breath with AutoPEEP that are not detected. These four values *TP*, *FP*, *TN*, and *FN* form the so-call confusion matrix of the detection. In terms of the four aforementioned evaluation measures, the performance results for the two proposed detectors are reported in Table 2. The results show that both the detectors worked very well on patient data with an accuracy higher than 93%, a precision higher than 99%, a recall (sensitivity) higher than 90% and a specificity higher than 98%. For the considered dataset, the two proposed AutoPEEP detectors provided similar results. It is worth mentioning that, by reducing the noise impact, the Sequential SNT-based detector is aimed at improving the detection performance of the Single-breath detector in case the latter fails to reveal ‘twilight region’ AutoPEEP, i.e. AutoPEEP with an end-expiration flow value near the given tolerance *τ*. Thence, the higher the number of twilight-region AutoPEEPs in the dataset, the more significant the performance improvement can be observed. However, in the considered clinical dataset, the number of twilight region AutoPEEPs, which are also difficult for the clinician to analyze, was very limited. Therefore, no significant difference in detection performance could be seen. However, the use of the Sequential SNT-based detector is recommended for better performance and robustness.

**Table 2:** Detection performance with flow data from patients

**Measure**	**Single-breath SNT-based detector**	**Sequential SNT-based detector**
Accuracy	**93.09%**	**93.09%**
Precision	99.44%	99.37%
Recall	**90.53%**	**90.60%**
Specificity	98.86%	98.70%

The experiments were carried out with *τ *= 2[l/min]. For both the detectors, the level was set to *γ *= 0.01, which corresponds to an average of 1 false-alarm per 5 minutes (with the usual breathing frequency of 20 [breaths/min]).

## Conclusion

To the best of our knowledge, this is the first work on the automatic detection of AutoPEEP for continuous monitoring of the patient-ventilator interface during controlled mechanical ventilation. With the introduction of the waveform vector to aggregate multiple samples into a unique decision, the SNT has been successfully applied to provide a good AutoPEEP detector. Finally, we have extended SNT in a sequential framework, namely *Sequential SNT*. The resulting sequential AutoPEEP detector has been shown to yield high detection performance. Besides, the proposed algorithms have very low complexity and require very little computational power. The platform can then be deployed as a real-time functional block.

Although the algorithm is proposed for the detection of AutoPEEP during controlled mechanical ventilation, it could be extended to assisted mechanical ventilation and pressure support ventilation since the algorithm investigates the expiratory part of the flow curve, which mainly depends on characteristics of the patient rather than on the ventilatory settings and mode of ventilation. The platform may also be extended to the detection of other types of ventilatory abnormalities that are deviations of the observed signal from some reference. In this respect, other signals such as pressure and volume curves could also be taken into account.

For the present work, by using the retrospective data files with a double-blinded and dual expert analysis, we were able to assess whether the system automatic analysis was concordant with that of the experts. In the next validation step, continuous and prospective recordings of the curves will be carried out to get better insight into cases where any disagreement between the proposed system and the therapist might occur. Furthermore, it is also worth performing a semi-closed-loop analysis, in which the therapist supervises, validates the decisions yielded by the proposed platform and adjusts the ventilatory parameters to correct any possible abnormality.

The deviation detection approach proposed in this paper is very general and could be used in many other applications, including fault detection and structural health monitoring. A theoretical general approach in Sequential SNT should also be investigated.

## Competing interests

The authors declare that they have no competing interests.

## Authors’ contributions

QTN and DP carried out the system design, algorithm development, implementation, and wrote the paper. DP proposed SNT and QTN worked on the adaptation of SNT to the problem and extended SNT in the sequential framework. ELH stated the medical issue, contributed to suggestions on the topic, provided data, clinical analysis, discussion and manuscript writing. All authors read and approved the final manuscript.
